# Performance of Coating Mortars with Partial Replacement of Natural Aggregate by Açaí Seed Residual Ash from the Amazon Region

**DOI:** 10.1021/acsomega.5c06658

**Published:** 2026-04-21

**Authors:** Bruno Lôbo de Almeida, Isaura Nazaré Lobato Paes, Luciana De Nazaré Pinheiro Cordeiro

**Affiliations:** Institute of Technology, Federal University of Pará, 01 Augusto Corrêa St., Guamá, Belém, Pará 66075-110, Brazil

## Abstract

This study evaluated external rendering mortars with partial replacement of natural sand by açaí seed residual ash (ASRA). Initially, through a pilot study, mortars containing 0, 10, 20, and 30% ASRA were tested, analyzing their properties in both fresh and hardened states. Considering the demand for sustainable solutions for agro-industrial residues, this research aims to corroborate, with technical and scientific evidence, the feasibility of using ASRA as a substitute for sand in mortars. Based on the pilot study analysis, it was found that the mixture with 20% replacement (ASRA20) exhibited the best rheological and mechanical performance. Subsequently, an external rendering mortar with 20% ASRA was produced and compared, in terms of performance, with a reference mortar widely used in Brazil (plasticized mortar without ash). Both mortars (REF and ASRA20) were applied to vertical masonry panels built with ceramic blocks, where the following aspects were assessed: cracking, surface friability, permeability, and direct tensile bond strength. In addition, microstructural analyses were carried out using scanning electron microscopy (SEM) and mercury intrusion porosimetry (MIP). The results, analyzed through statistical methods, demonstrated that the ASRA20 mortar presented improved particle cohesion, higher plasticity, a lower cracking index, greater resistance to surface wear (scratch test), reduced permeability, and bond strength values exceeding the requirements established by standards. These findings indicate that ASRA has strong potential for application in cementitious matrices, contributing to the reuse of an abundant residue from the Amazon region and providing a sustainable alternative for construction materials.

## Graphical abstract

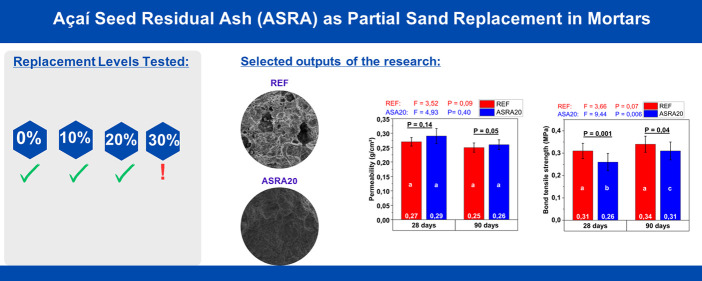

## 1 Introduction

Açaí production in Brazil has grown significantly, from 0.22 million tons in 2015 to 1.58 million tons in 2023, representing more than a 7-fold increase. The Amazon region is responsible for the majority of this production, mainly concentrated in the state of Pará, which accounts for approximately 92.9% of the total volume, generating a value of BRL 8.06 billion, according to 2023 data.^1^

In the açaí pulp extraction process, the yield varies between 5% and 29%, resulting in a waste output of 71% to 95% (by mass), composed primarily of seeds and fibers.^2,3^ This high volume of discarded material poses a major environmental challenge. In 2023, for instance, this waste amounted to a minimum of 1.12 billion tons, based on IBGE data.^1^

Açaí seeds have a fixed carbon content between 21.39% and 21.63%, with a high calorific value ranging from 19.32 MJ.kg^–1^ to 19.91 MJ.kg^–1^.^4^ Due to these characteristics, the residue emerges as a viable alternative for energy generation in agro-industries and as a potential fuel for boilers and ceramic factories, replacing firewood and coal.^5–7^

However, using this biomass as fuel generates a new waste: açaí seed residual ash (ASRA). This ash presents high heterogeneity because its combustion process is not controlled, which compromises its pozzolanic activity according to the criteria.^8^ Nevertheless, this does not preclude its use in cementitious mixtures, since it can contribute to matrix packing due to its physical characteristics, assisting in void filling through the filler effect.

In this context, açaí seed ash has been the subject of research for its incorporation into cementitious matrices as a partial replacement for fine aggregate or cement. The use of this material has shown promising results, both in the fresh and hardened states. Studies indicate that the inclusion of ash promotes changes in the microstructure of concrete and mortar, favoring more efficient particle packing and resulting in a more cohesive structure.^9^

In this regard, a study evaluated the influence of açaí seed ash on the properties of roughcast mortars and found, among other results, that mortars containing 10% açaí seed ash showed reduced air content and shrinkage, as well as increased compressive strength.^10^

Another study on the use of açaí seed ash as a mineral additive in structural concrete, which was subjected to accelerated carbonation tests.^11^ The authors concluded that the concrete containing the ash exhibited a lower carbonation index. Likewise, a study, also evaluating the incorporation of this type of ash in concrete, reported increased density and satisfactory physical and mechanical performance.^12^

Given the above, this study aimed to evaluate the performance of mortars using açaí seed residual ash (ASRA) as a partial replacement for natural fine aggregate, at three different replacement levels. The research involved the characterization of the ash, analysis of the mortars in fresh and hardened states, and performance evaluation of the coatings when applied to ceramic block masonry.

It is also important to highlight that the use of ash as a partial replacement for sand in rendering mortars comes at a critical time, as the construction industry model practiced in Brazil, throughout its production chain, causes several environmental impacts. It relies heavily on nonrenewable natural raw materials and consumes a high amount of energy in the extraction, transportation, and processing of inputs.

In 2015, the United Nations established 17 Sustainable Development Goals (SDGs) across various areas to be achieved by 2030.^13^ Among these, this study aims to contribute to at least three:

- SDG 9 (Industry, Innovation and Infrastructure): Promotes innovation in the construction sector by introducing agro-industrial waste as input, encouraging sustainable practices;
- SDG 11 (Sustainable Cities and Communities): Contributes to more sustainable cities by reducing the consumption of natural resources and promoting waste reuse;
- SDG 12 (Responsible Consumption and Production): Supports the circular economy by reusing a waste material that would otherwise be discarded, thereby reducing environmental impacts.

Thus, this research not only evaluates the performance of mortar with natural aggregate replacement, but also contributes to sustainable development and the valorization of an abundant residue in the Amazon region, transforming it into a coproduct that reduces environmental liability and stimulates the regional economy through socioeconomic benefits.

## 2 Materials and Methods

### 2.1 Materials

For the production of the reference mortar, as well as for those containing partial replacement of sand with açaí seed residual ash (ASRA), the following materials were used: Portland cement CPV-ARI, natural aggregate, açaí seed residual ash, plasticizer additive, and ceramic masonry blocks, which were used to prepare panels for the application of the mortars and to evaluate their performance as a system. In addition, potable water at a temperature of 28 °C and pH 6 was used, supplied by the local utility company and compliant with the Brazilian standards for use in cementitious materials.^14^

Portland cement CPV-ARI (high early strength Portland cement) was selected for the mortar compositions due to its purity, as it contains no mineral additions. Regarding its physical characteristics, the material presented a 75 μm sieve residue of 0.2%, specific surface area of 438.6 m^2^/kg, average particle diameter of 9.16 μm, specific gravity of 3.10 g/cm^3^, initial setting time of 150 min, and final setting time of 210 min. Its compressive strengths were 34.7, 41.1, and 44.7 MPa at 3, 7, and 28 days, respectively, in accordance with by the Brazilian standard.^15^ The chemical characteristics of this cement are presented in Table 1, along with those of the ash.

**Table 1:** Chemical Characterization of the Cement and Açaí Seed Ash, Source: Cordeiro et al., (2019)

**CHEMICAL COMPOSITION OF CEMENT (%)**									
**C**_**3**_ **S**	**C**_**2**_ **S**	**C**_**3**_ **A**	**C**_**4**_ **AF**	**CaSO**_**4**_	**MgO**	**SO**_**3**_	**CO**_**2**_	**RI**	**PF**
66,86	15,74	6,16	9,89	5,18	1,73	3,05	1,18	0,41	2,85

**CHEMICAL COMPOSITION OF RESIDUAL AÇAÍ SEED ASH (%)**								
**SiO**_**2**_	**Al**_**2**_ **O**_**3**_	**Fe**_**2**_ **O**_**3**_	**CaO**	**MgO**	**P**_**2**_ **O**_**5**_	**Na**_**2**_ **O**	**K**_**2**_ **O**	**PF**
35,33	7,18	0,79	4,57	4,13	12,67	0,34	18,61	14,23

The residue, originating from açaí seed residual ash (ASRA), was collected from kilns of ceramic factories located in the municipality of Castanhal, in the state of Pará (PA), Brazil. It is noteworthy that the açaí seeds initially undergo an air-drying process to remove surface moisture and are subsequently fed into the kilns as a substitute for natural wood firewood. These kilns do not operate under controlled temperatures, resulting in a highly heterogeneous residue that, to date, has not been reused in the region.

The characterization of the açaí seed residual ash (ASRA) was carried out through X-ray fluorescence (XRF), loss on ignition (LOI) testing, and scanning electron microscopy (SEM) imaging. The results obtained are presented in Table 1 and Figure 1.

**Figure 1:**
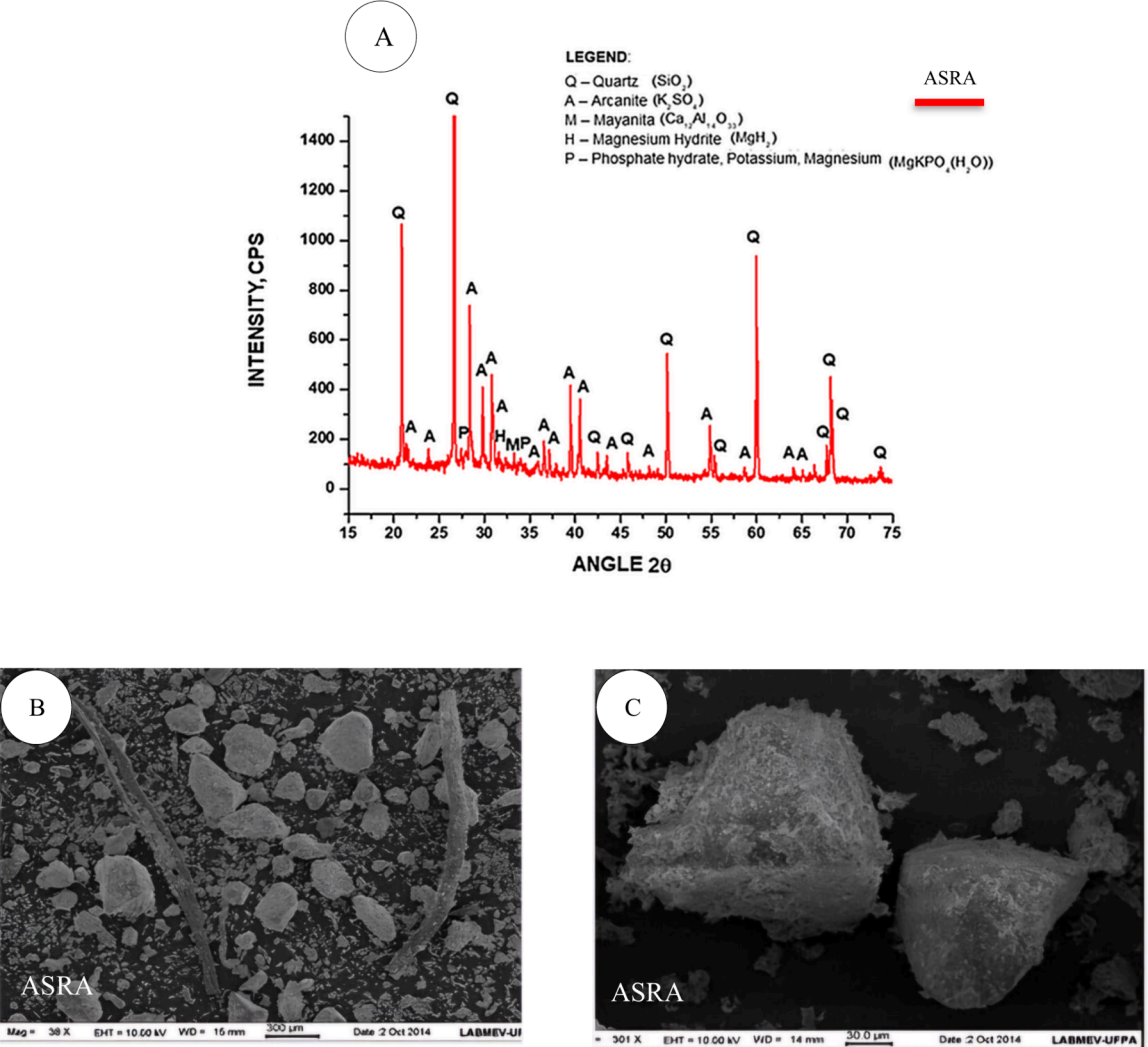
Physical and morphological characteristics of residual açaí seed ash (ASRA) and sand: X-ray diffraction of ASRA (A); microstructure obtained by scanning electron microscopy of ASRA (B and C) Source: Figure 1. Adapted with permission from Cordeiro, L. de N. P.; Paes, I. N. L.; Souza, P. S. L.; Azevedo, C. M. Caracterizacao da Cinza de Caroco de Acai Residual para Adicao ao Concreto. Ambiente Construído 2019, 19(1), 45–55. Copyright © 2019 Associacao Nacional de Tecnologia do Ambiente Construido (ANTAC).

Regarding the X-ray diffraction (XRD) pattern shown in Figure 1A, the material exhibits some crystallinity peaks and the presence of calcite, likely due to contamination. The material does not present high reactive potential, which may hinder pozzolanic activity. Therefore, the ash was used with the aim of evaluating its ability to improve the microstructure through the filler effect.

With respect to its micromorphology, shown in Figure 1B,C, the residual ash exhibits an irregular shape, with some well-defined edges and varying particle sizes. Its surface shows a certain roughness and irregularities, which may be the result of the grinding process. Additionally, in Figure 1B, particle agglomeration can be observed, which indicates heterogeneity due to nonuniform distribution and may negatively affect the final performance of the material.

The natural aggregate used was river sand, with a specific gravity of 2,630 kg/m^3^, bulk density of 1,520 kg/m^3^, fineness modulus of 1.75, and a maximum particle size of 1.2 mm.

The plasticizing additive used in this study is a nationally produced product with an organo-synthetic composition. Its main active components are byproducts from the distillation of pine resin and rosin. The additive is commercialized in liquid form, with a dark color, a pH of 10.5, and a density of 1.1 g/cm^3^. It contains 5% solids by mass and is fully soluble in water. According to the manufacturer’s information, the recommended dosage is 200 mL per 50 kg bag of cement.

The aggregate used was a natural quartz sand, which was subjected to tests to determine its physical characteristics, as presented in Table 2 and Figure 2.

**Table 2:** Characterization of Fine Aggregate – Natural Sand

**Determined Characteristic**	**Test Method**	**Results**
**Maximum Diameter (mm)**	ABNT NBR 17054:2022	1.2 mm
**Fineness Modulus**	ABNT NBR 17054:2022	1.58
**Bulk Density** (g/cm^3^)	ABNT NBR 16972:2021	1.73 g/cm^3^
**Specific Gravity** (g/cm^3^)	ABNT NBR 16916:2021	2.67 g/cm^3^
**Average Swelling Coefficient**	ABNT NBR 6467:2006	1.72
**Critical Moisture (%)**	ABNT NBR 6767:2006	2.60%
Void Index = [1 - (BD/SG)] × 100	-	35%

**Figure 2:**
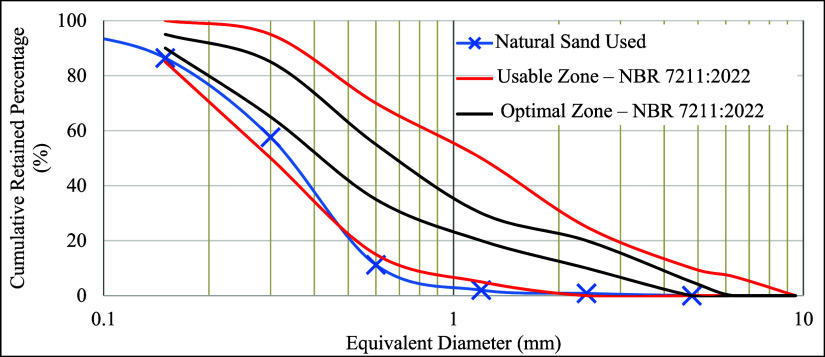
Sand particle size distribution curve. Source: Autor.

Figure 2 shows the particle size distribution curve of the fine aggregate, along with the recommended usage limits according to standard.^16^ It can be observed that the material does not fall within the optimal zone but is mostly contained within the usable zone, except for the smaller particle sizes. Thus, it is classified as a fine sand, with a high swelling coefficient (72%) and low critical moisture.

It is noteworthy that this type of granulometry (uniform and small-sized particles) is common in the sands where this study was conducted and naturally leads to higher water demand in the mortar mix due to the large specific surface area of the grains.

For the design of the plastered coatings, mortars were applied over masonry panels made of six-hole ceramic blocks, whose characterization parameters are presented in Table 3.

**Table 3:** Characterization of the Blocks, According to the Brazilian Standard^17^

**Determined Characteristics**	**Test Method**	**Number of Determinations**	**Average Results**	**Coefficient of Variation (%)**
**Water Absorption (%)**	MB-3459 (ABNT, 1991)	12	19.6%	11.16
**Initial Rate of Absorption (IRA)**	ASTM C-67	12	11.6 g/194 cm^2^/min	14.40
**Compressive Strength**	MB-3459 (ABNT, 1991)	12	0.56 MPa	16.20
**Dimensions**	MB-3459 (ABNT, 1991)	12	height = 13.5 cm,width = 9.0 cm,length = 19.0 cm	13.18

The blocks exhibited a high total water absorption capacity, practically at the limit established by Brazilian standards, which is 20%.^17^ The increasing curve, with no significant distinction between the initial and final absorption regimes, tends to affect the performance of the coatings, particularly the bond strength, due to the blocks’ capacity to absorb hydration products from the mortars while still in the plastic state.

### 2.2 Methods Employed

#### 2.2.1 Pilot Study

The starting point for the development of mortars incorporating açaí seed residual ash was the implementation of a pilot study, as shown in Figure 3.

**Figure 3:**
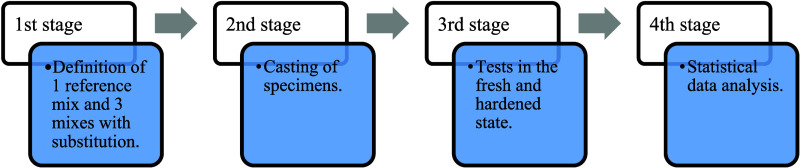
Stages of the pilot study. Source: Autor.

Mortars were developed with partial replacement of sand by açaí seed residual ash (ASRA) in the following proportions: 0% (reference), 10%, 20%, and 30%. These replacement levels were selected based on previous studies in the literature, which indicate that such ranges are suitable for achieving acceptable performance when using agro-industrial ashes. Examples include research in which satisfactory results were obtained within this range for both fresh and hardened states.^18,19^

A reference rendering mortar commonly used in the metropolitan region of Belém/PA was adopted, with a mix ratio of 1:5 (cement:sand, by volume) and a plasticizer admixture. The admixture dosage was 200 mL per 50 kg of cement. After the admixture was added, the mixing time was limited to 5 min in order to avoid excessive air incorporation. The influence of the varying ASRA replacement levels was evaluated through characterization tests in both fresh and hardened states, following the Brazilian standards: consistency;^20^ water retention;^21^ bulk density and air content;^22^ compressive and flexural strength;^23^ and water absorption.^24^

The batching, molding, and curing stages of the specimens were performed according to ABNT NBR 5738.^25^ Mortars were produced using a tilting drum mixer with a capacity of 150 L and a rotation speed of 28 rpm. Immediately after mixing, tests were conducted on the fresh mortar and samples were prepared for hardened state evaluation. Demolding took place after 24 h, after which the specimens were placed in a moist curing chamber until testing. The mix proportions (by mass) are presented in Table 4.

**Table 4:** Material Consumption Used in the Production of Rendering Mortars

			**Material quantities** (kg/m^3^)			
**Substitution (%)**	**Mass mix ratios** cement:ASRA:sand	Water/Cement ratio (w/c)	Cement	ASRA	Sand	Water
0% **(REF)**	1:0:4,30	1,07	328	-	1410	351
10% **(ASRA10)**	1:0,30:3,86	1,20	328	98	1265	393
20% **(ASRA20)**	1:0,58:3,45	1,29	328	190	1131	423
30% **(ASRA30)**	1:0,86:3,00	1,33	328	282	983	436

#### 2.2.2 Performance Evaluation of Mortars Applied on Vertical Panels

From the pilot study, two mortar mix designs were selected for application on vertical panels built with ceramic block masonry: the reference mortar (REF, with 0% substitution) and the mortar with 20% replacement of fine aggregate by the ash (ASRA20). These mixes were chosen due to their superior performance in the pilot tests, based on the characteristics observed in both fresh and hardened states. Based on these definitions, the research proceeded with the experimental steps shown in Figure 4.

**Figure 4:**
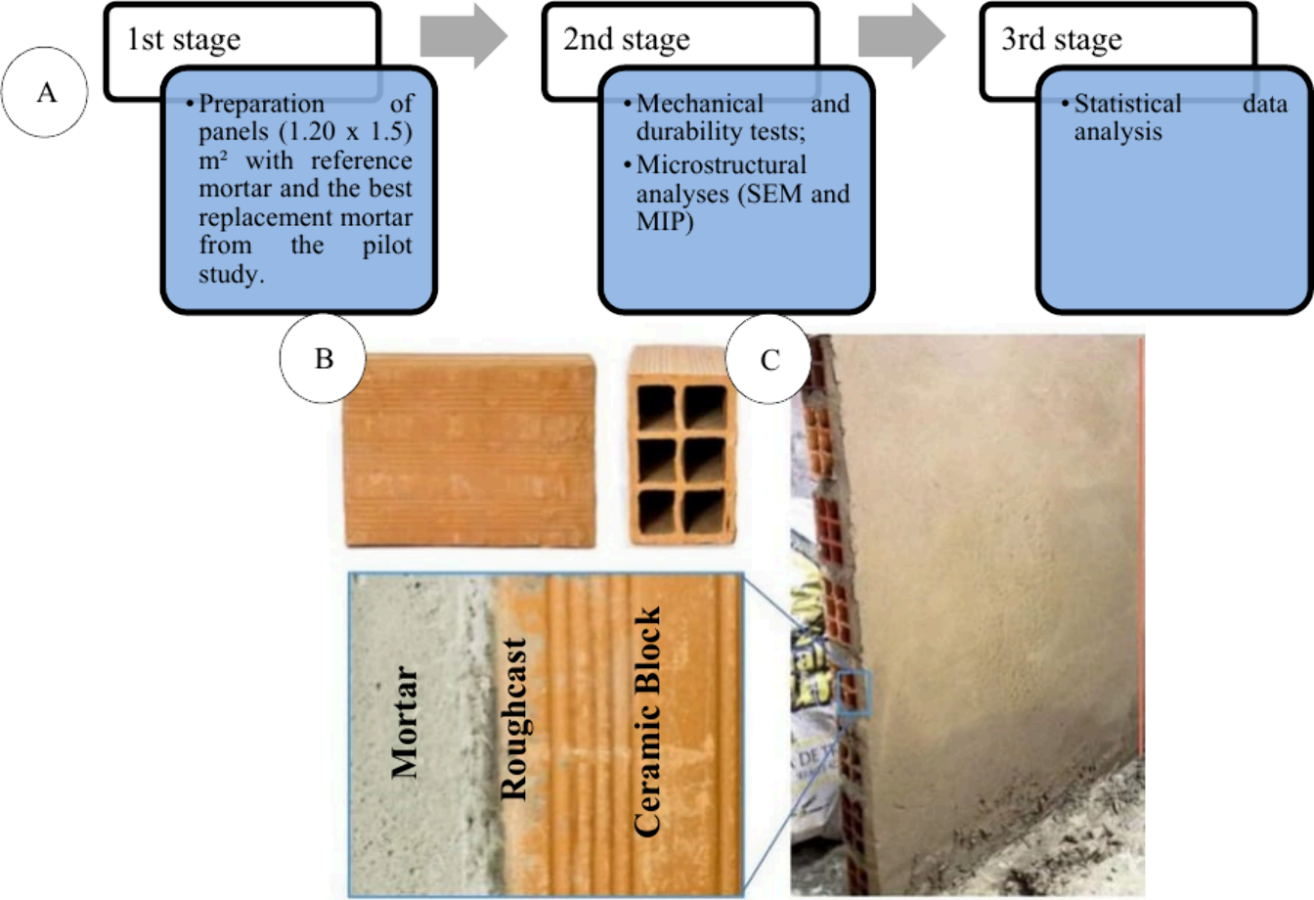
Phases of the mortar panel study. Source: Autor.

The mortar application was carried out on four vertical panels made of six-hole ceramic blocks, each measuring 1.20 m × 1.50 m (Figure 4C). These were built in an outdoor area in order to expose the coatings to real environmental weathering conditions during the research. After the construction of the panels, they were roughcast (a base treatment mortar with a rough texture), using a recommended 1:3 mix ratio (cement:sand, by volume), applied with a mason’s trowel.^26^ Curing (a humidification procedure aimed at enhancing hydration of the cement matrix) was carried out by water spraying for 3 consecutive days, four times per day. Fourteen days after the roughcasting, the panels were coated with the REF and ASRA20 mortars.

After the mortars hardened, a visual inspection was performed to evaluate the finished surface for cracking and surface abrasion resistance (powdering). The latter was assessed as follows: after mortar application, at early ages (3, 7, and 14 days) and later ages (28 and 90 days), a sharp metallic object was pressed against the coating surface, following the recommendations of studies.^27^ In their study, the authors suggest observing the depth of penetration—where a deeper mark indicates lower surface hardness and resistance.

As for cracking, its appearance and development were monitored on each panel up to 90 days. Cracks were classified based on their width using a magnifying glass and a crack width gauge. Lateral markings were made to track their progression, if any. Quantification was done by summing the measured crack lengths (in millimeters) and dividing by the area of each panel, yielding a value per square meter (m**^2^**). Cracks near the panel edges were disregarded as they likely resulted from imperfections during finishing.

The permeability of the coatings was assessed using the method proposed by the *Centre Scientifique et Technique de la Construction*,^28^ known as the “pipe method”. This method uses L-shaped glass tubes (Karsten tubes), graduated from 0 to 4 mL (1 mL = 1 cm^3^), which are fixed to the test surface with elastomeric sealant. When filled to the top, the water column exerts a pressure equivalent to 98 mm water head, simulating wind pressure of around 40 m/s (≈140 km/h) perpendicular to the surface. Readings can be taken at 5, 10, 15, 30, and 60 min, or until 4.0 cm**^3^** is absorbed. For in situ tests, this duration may be reduced to 15 min, with measurements taken every minute. In this study, results are presented for 5, 10, and 15 min.

The tensile bond strength of the coatings was evaluated according to the minimum values prescribed by the Brazilian specification standard.^29^ Tests were performed at 28 and 90 days, following procedures from Brazilian Standard, which prescribes the use of a pull-off tester (impact dynamometer).^30^ In this research, 12 pull-off tests were conducted per panel and per mortar type, totaling 48 measurements. Recorded data included failure loads, effective specimen diameters, coating thickness, and types of failure.

At 90 days, samples were extracted for Scanning Electron Microscopy (SEM) imaging and Mercury Intrusion Porosimetry (MIP) to assess the pore size distribution of both mortars.

SEM was used to observe surface morphology at 90 days, providing high-resolution images from the interaction of secondary electrons with the sample, revealing topographical and compositional details.

MIP was employed to investigate microstructural differences and pore size distribution caused by the inclusion of açaí seed residual ash in the mortar, as compared to the reference mix and considering water loss due to suction from the porous ceramic block substrate. Despite its limitations (e.g., inability to access closed pores), the method is valid for comparing materials of similar nature.^31^ Porosity is a key morphological property affecting durability and, consequently, sustainability.^32^

The test used a Micromeritics Autopore III 9410 porosimeter. Test specimens were ≤8 mm in diameter and 25 mm long, extracted from the central region of the coatings to avoid carbonation. They were predried to eliminate gases and vapors before mercury injection, via two steps: immersion in technical-grade anhydrous isopropanol for 10 days, followed by vacuum drying at 100 °C for 20 h.

Testing began with a vacuum pressure of 50 μmHg for 5 min, then mercury was injected. The initial pressure was approximately 0.798 psi, increasing to a maximum of 32,611.775 psi. A contact angle of 140° and mercury surface tension of 0.48 N/m were used.

Results were expressed as (a) Mercury Volume vs Pore Diameter curve – indicating the quantity of pores of a given diameter by the volume of mercury intruded; (b) Cumulative Mercury Volume vs Pore Diameter curve – indicating the total mercury volume per unit mass intruded at a given pressure, reflecting porosity up to the corresponding pore diameter. Curves represent the average of two samples tested for each mortar (REF and ASRA 20).

## 3 Results and Discussion

Fresh and hardened state properties of ASRA mortars were compared with the reference mix. In addition, a comparative performance test was carried out between the reference mortar and the mortar containing the ash with the best performance observed in the pilot study. These two mortars (REF and ASRA20) were subsequently applied onto ceramic brick masonry.

In the initial stage of data processing, normality and homoscedasticity were verified. The Shapiro-Wilk test was applied to assess normality, adopting a significance criterion of *P* > 0.05. The homogeneity of variances was evaluated using Levene’s test, also considering *P* > 0.05. Based on these results, statistical analysis proceeded with the application of posthoc tests. Data were presented as arithmetic mean ± standard deviation, with significant differences considered when *P* < 0.05.

### 3.1 Evaluation of Mortar Characteristics in the Fresh State Based on Observations from the Pilot Study

In the fresh state, the following tests were carried out: consistency, air content, bulk density, vane test, and water retention. The results are shown in Figure 5A–D, through which correlations between the test outcomes are explored.

**Figure 5:**
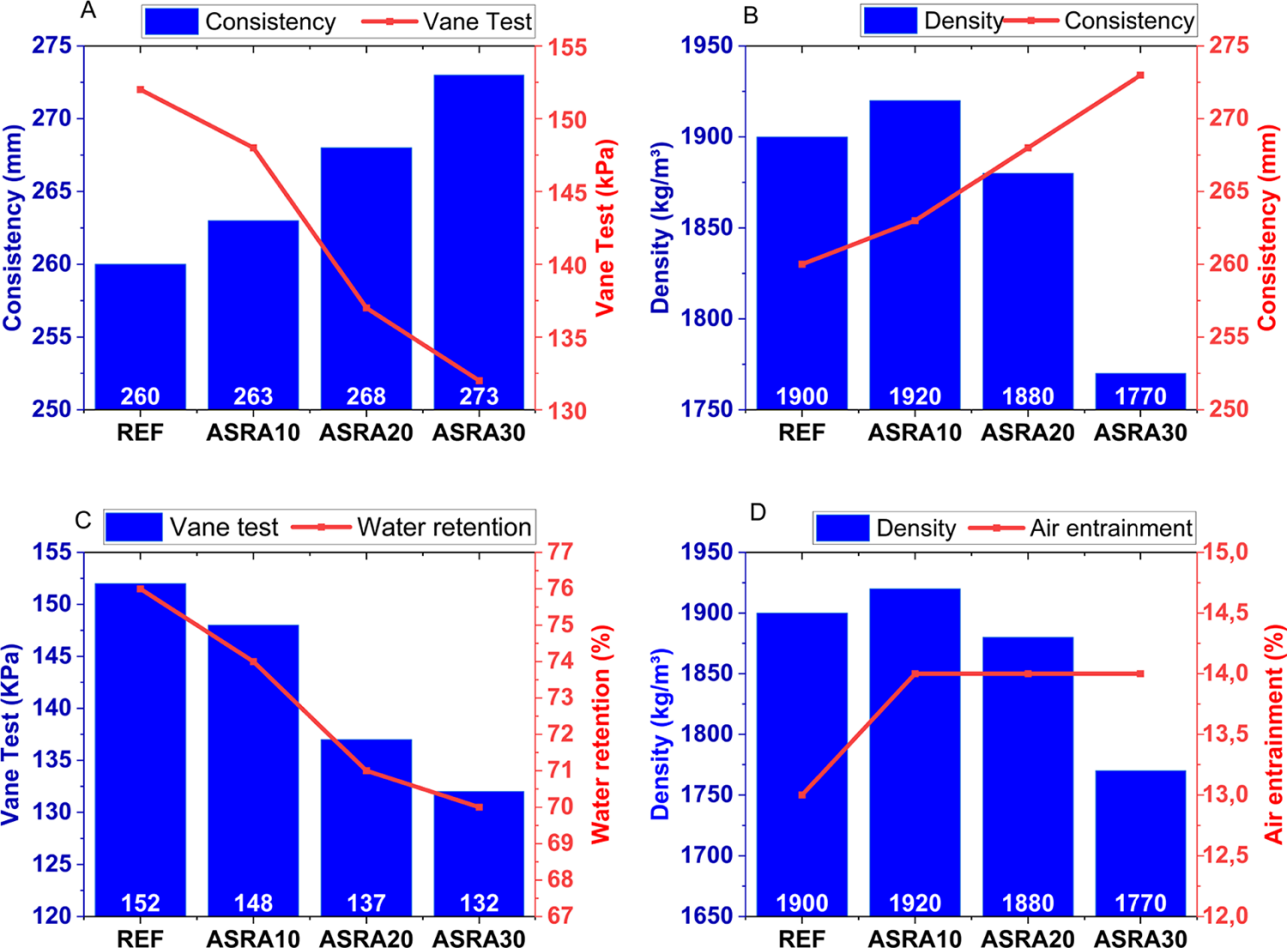
Fresh mortar tests: (A) consistency vs vane test; (B) density vs consistency; (C) vane test vs water retention; (D) density vs air content. Different lowercase letters indicate significant differences within the same variable. For clarity, standard deviations are not shown in some graphs, all below 5%. Source: Autor.

Figure 5A shows that the presence of ASRA results in an increase in consistency and density (except for ASRA30), along with a reduction in shear strength (Figure 5C). These factors are important, as they indicate a mortar that is workable for the mason.

With increasing ash content, a higher amount of water was required to ensure the plasticity of the mixtures. This can be attributed to the increased specific surface area of the constituent materials (cement, sand, and ash), which demanded higher liquid content, since there is generally a direct relationship between the content of fine particles and water demand.^33^

Air entrainment, however, remained practically unchanged, with values around 14% for all three ash contents. One reason for this stability was the strict control of the admixture dosage (in accordance with the manufacturer’s recommendations) and mixing time (5 min), which aimed to enhance plasticity without excessive air incorporation. In general, the literature reports that the amount of entrained air is inversely proportional to density.^34^ In this study, the variation in air content and density was not significant, which is beneficial, as excessive air incorporation strongly influences mechanical strength, permeability, and durability of concretes and mortars, particularly with respect to fluid transport through their internal structure.^35–37^

The spherical microbubbles generated in the fresh state are uniformly distributed within the cementitious matrix and may create a network of impermeable (nonconnected) pores, directly reducing component permeability. On the other hand, excessive bubble formation may reduce the effective bond area between the mortar and the substrate, as too much entrained air can hinder the transport of moisture from the plastic mortar to the porous base—a fundamental process for both physical and mechanical anchorage of the coating and the formation of cement hydration products.^32,38–41^

From qualitative tactile and visual observations, the following aspects were noted: the mortar with 10% ASRA did not provide satisfactory plasticity, suggesting the need for the addition of an auxiliary agent, such as lime or admixture. The mortar with 20% ASRA exhibited high workability, indicating that this level was close to an “optimal value”. Conversely, the 30% ASRA mix was perceived as “rough and heavy,” requiring adjustments for practical application, similar to ASRA10%.

This behavior can be explained by the fact that the ash does not exhibit plasticizing/surfactant or hydrophilic properties. Its highly porous microstructure, combined with the presence of organic material due to uncontrolled burning, produces a higher amount of fibrillar pores, which promote water loss and reduce retention, giving the mortar a “rough” aspect.^42^

### 3.2 Evaluation of Hardened State Characteristics Based on Observations from the Pilot Study

For the hardened state tests, the mechanical property results were analyzed by comparing the REF mortar with the other treatments. Figure 6A–D shows the results of the tests, aiming to explore the relationships between the variables.

**Figure 6:**
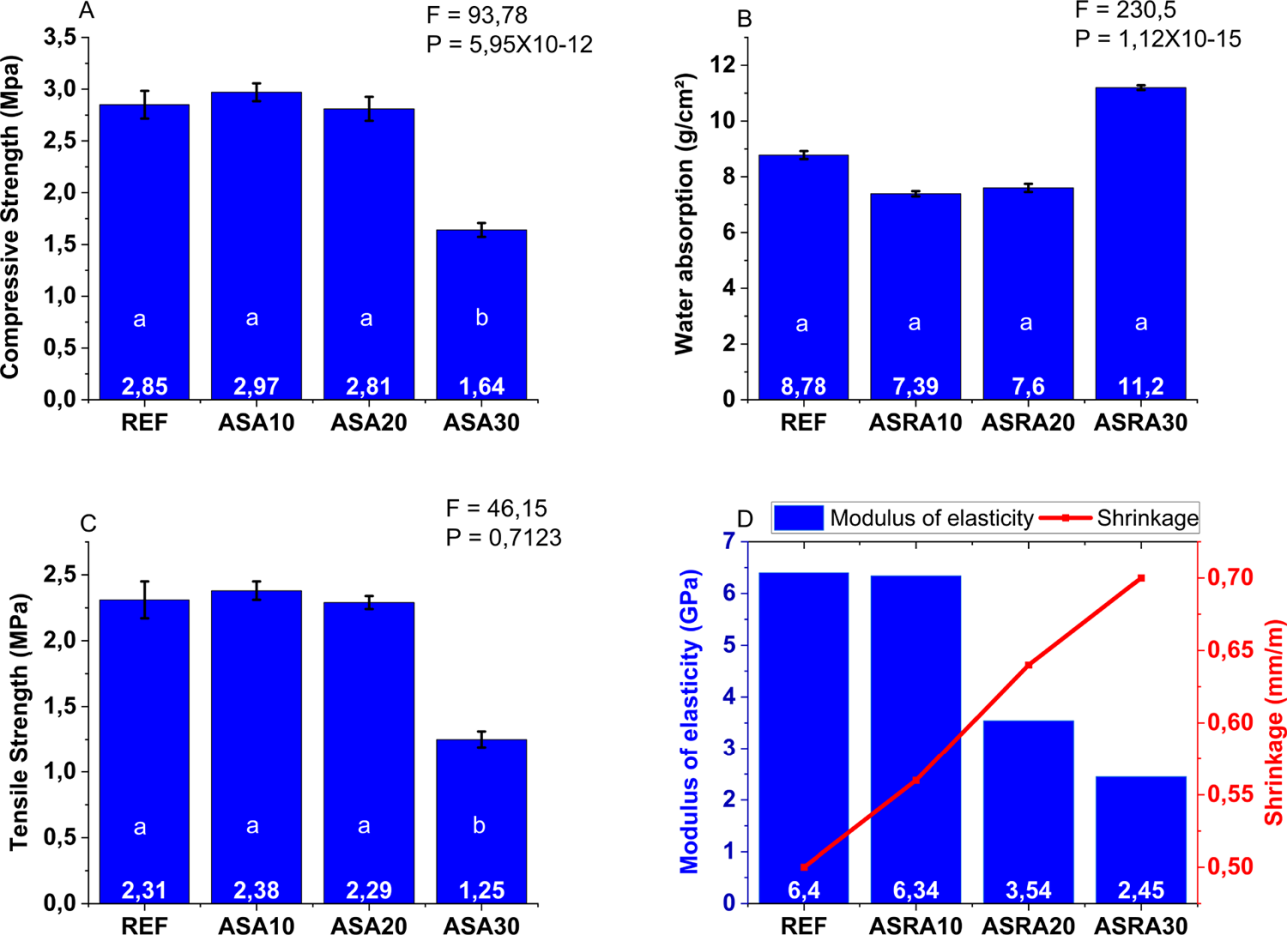
Hardened mortar tests: (A) compressive strength; (B) water absorption; (C) tensile strength; (D) modulus of elasticity vs shrinkage. Values are expressed as mean ± standard deviation. Different lowercase letters indicate significant differences within the same variable. For clarity, standard deviations are not shown in some graphs, all below 5%. Source: Autor.

Based on posthoc tests in the axial compression and flexural tensile strength tests, only the treatment with ASRA30 showed a significant difference compared to the reference (REF), with a reduction in the average axial compressive strength. Thus, statistical analysis demonstrated that, as observed in the fresh state, the ASRA30 mortar exhibited significant differences in relation to the reference mortar. The mechanical strengths in axial compression and flexural tensile strength decreased by approximately 43% and 46%, respectively, when comparing REF and ASRA30 mortars. As for total water absorption of the mortars, no significant differences were observed.

The increase in the water-to-cement (w/c) ratio from 1.07 (REF) to 1.33 (ASRA30) explains the lower early age strength and greater shrinkage observed, since excess water promotes higher porosity in the hardened matrix. As previously noted, the ASRA10 and ASRA20 mortars, although requiring more water, did not show significant variations in strength compared to the reference mortar (without ash addition). This increase in w/c is attributed to the incorporation of ash particles with high specific surface area and internal porosity, which tend to aggregate (coalesce) due to van der Waals and capillary surface forces. Such coalescence reduces the effective dispersion of particles in the paste, increasing the formation of agglomerates that retain water internally and intensify interparticle friction.^43^ Consequently, more water is required to break down these agglomerates and restore the desired mortar fluidity.

This behavior is widely reported in the literature, highlighting that higher w/c ratios lead to a more porous microstructure and, consequently, inferior mechanical performance at early ages.^44^ On the other hand, prolonged hydration promotes pore refinement, partially compensating for these drawbacks. Recent studies confirm that although pastes with high water content exhibit elevated initial porosity, a significant reduction in pore volume occurs with the progress of hydration at later ages, indicating positive microstructural evolution.^45^ Complementary reviews also point out that long-term hydration and rehydration can improve mechanical properties and reduce effective porosity, corroborating that early performance losses tend to be partially compensated at advanced ages.^46^

ASRA30 was the only mortar with a higher water absorption result than the reference mortar (27% higher). This test allows for the observation of water movement through capillaries, which are pathways formed by the interconnection of pores and may act as a natural barrier. Therefore, the materials that compose the mortar, especially the presence of additions (such as ASRA) and chemical admixtures (such as plasticizers), are key factors in this moisture percolation process.^39,40,47^ In this regard, it can be seen that the pore network formed from the composition of ASRA30 likely exceeded the optimal ash content, and consequently, led to undesirable characteristics in this mortar, both in the fresh state (lack of plasticity) and in the hardened state (high water absorption and reduction in mechanical properties).

The strength results show that the treatments with 10% to 20% replacement represent the optimal range for partial substitution of sand with ASRA, showing strength levels similar to the reference mortar. This may be explained by the particle size distribution of the sand, combined with the ash, having resulted in better packing and, consequently, increased strength, possibly due to a reduction in void volume.

The exception was the ASRA30 mortar, which did not show improvements in its mechanical properties compared to the reference mortar. The packing of the mixture negatively affected its performance, as shown by the plastic shrinkage results. As expected, replacing a high percentage (30%) of the coarser aggregate with finer residual ash required more water, which in turn increased the water-to-cement ratio, creating a “granular skeleton” with more interstices and greater plastic shrinkage.

Therefore, overall, the 20% ASRA mortar presented the best plasticity characteristics, satisfactory mechanical strengths, and the lowest water absorption values, even when compared to the reference mortar. Thus, Table 5 presents a brief summary of the mortar properties in the fresh and hardened states that were considered for selecting the material to be evaluated in wall panels. Based on these results, ASRA20 was chosen for performance assessment on vertical panels built with ceramic blocks, as it exhibited the best overall performance by combining the highest compressive strength (3.09 MPa), good tensile strength (2.29 MPa), low water absorption (7.6 g/cm^2^), and adequate consistency (268 mm). The lower modulus of elasticity compared to mixes with smaller ash contents may also help reduce stresses and cracking in the panels. Therefore, ASRA20 was considered the most suitable mixture for evaluation in vertical ceramic block panels. These results are consistent with findings from other studies that investigated partial sand replacement with agro-industrial ashes.

**Table 5:**
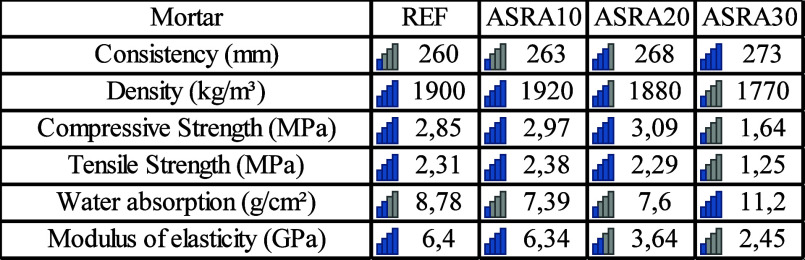
Summary of Mortar Properties in the Fresh and Hardened States

These results align with findings from other studies that replaced sand with agro-industrial byproducts.^19,48–51^ These studies show that the replacement of up to 20% of sand with agro-industrial ashes in cementitious compositions maintains strength levels comparable to those without substitution, while replacements over 25% generally lead to significant decreases in mechanical performance.

### 3.3 Performance and Durability Evaluation of ASRA20 Mortar Applied on Vertical Panels

It was observed that in the panels where the reference mortar was applied, the number of visible cracks was practically negligible, with an average width of 0.2 mm. These cracks likely would not significantly compromise the performance of the coatings in terms of penetration by aggressive agents, including rainwater, which is one of the main causes of pathological manifestations in the region where this research was conducted.^52^

In contrast, the panels with ASRA20 mortar showed about 10% more visible cracks, with an average width of 0.33 mm. This may be attributed to the higher water demand required to achieve adequate workability of this mortar. However, most of the observed cracks were superficial and classified as passive cracks, meaning they did not widen or deepen over time and were most likely due to drying shrinkage in the early ages.

Thus, it was noted that both the REF and ASRA20 mortars exhibited appropriate surface hardness and cohesion between particles, with an increase in these characteristics at later ages as the hydration reactions of the cementitious matrix progressed.

In this context, curing of the mortars is a key protection against premature drying, especially due to wind and solar radiation, which is essential in preventing cracking due to hydraulic shrinkage.^53^ Although curing does not reduce the final shrinkage value, it ensures that shrinkage occurs at a time when the mortars already possess sufficient mechanical strength, thereby reducing the incidence of cracking. As such, the continued hydration process and pore blocking tend to be reflected in the mechanical properties of cement-based materials, increasing the durability of mortar coatings.^54^

When it comes to building façades, a combined assessment of permeability and mechanical strength is essential, as the connection between these properties is undeniable—particularly regarding efficient coating systems that serve as protective envelopes for buildings and provide adequate conditions of health, watertightness, functionality, and comfort for users throughout the building’s service life.^55^

Therefore, water permeability is a property directly related to the watertightness of masonry and becomes especially important in façade coatings. Water can penetrate a given material, component, or construction element by infiltration under pressure, capillary action, or water vapor diffusion through capillary channels.^56–59^ In this sense, this research measured this property at two different ages (28 and 90 days). For each panel, three water absorption measurements were performed, all on the blocks themselves, avoiding measurements on the bed joints, as this region tends to be more porous. The permeability results of the mortars are presented in Figure 7.

**Figure 7:**
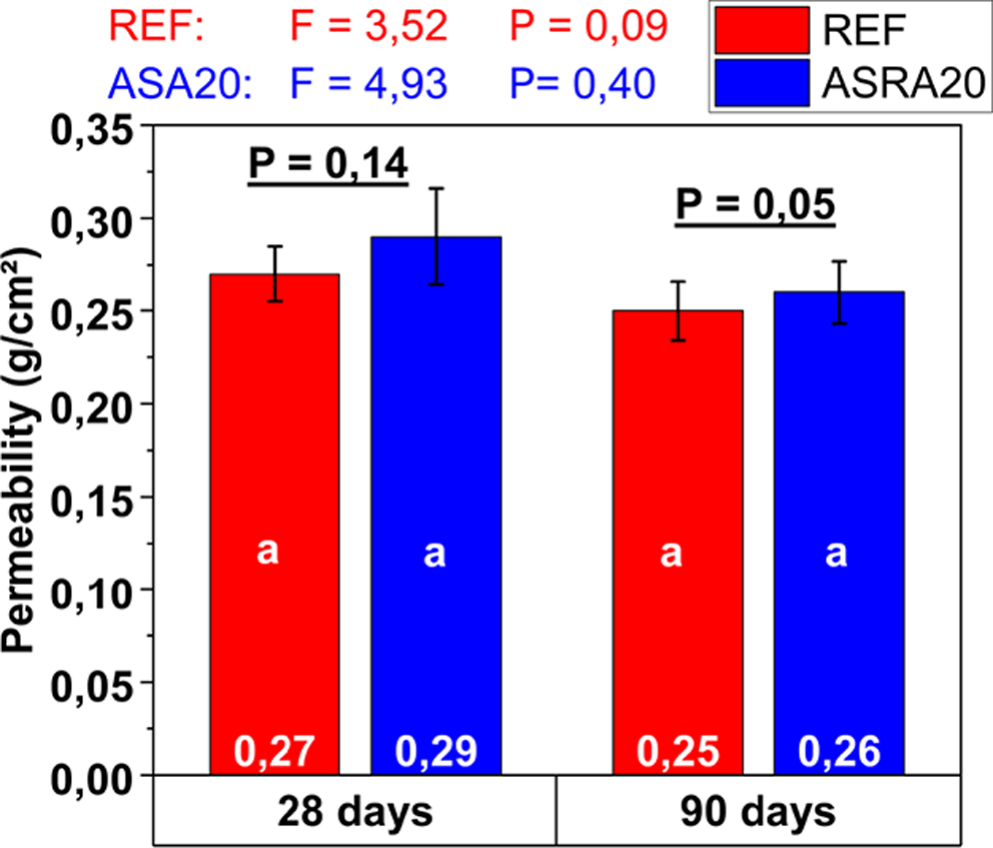
Water permeability of reference mortar and ASRA20 mortar at 28 and 90 days. Mean values ± standard deviation. Different lowercase letters indicate significant differences under the same variable. Source: Autor.

Initially, although the mortar with ash showed approximately 7% higher permeability than the reference mortar, there was no statistically significant difference in this property, regardless of the age evaluated, as shown in Figure 7.

At a more advanced age (90 days), a trend toward decreased permeability can be observed, as it varies with the progress of cement paste hydration. The literature explain that, as the hydration process and crystal formation advance, the total volume of gel fills part of the space initially occupied by water.^36,60^ After the early ages, the size, shape, and concentration of the gel particles, as well as the continuity or discontinuity of the capillaries, determine the material’s permeability.

In this context, it is important to highlight that the mortar used in this study contains a plasticizing admixture, which also has a significant secondary effect of air entrainment and promotes greater water retention.^35,61,62^ At the same time, this air entrainment tends to increase the mortar’s porosity, making it more permeable and susceptible to deterioration due to moisture and aggressive agents.^37^

Therefore, it is essential to control the dosage of these admixtures, which increasingly require a combined analysis considering the chemical bases employed,^63,64^ the type of cement,^65^ the aggregate’s particle size distribution,^66^ and the mixture’s characteristics,^37,39,67^ among other factors, in order not to negatively affect the permeability and strength of the rendering system. Accordingly, Figure 8 evaluates the tensile bond strength of the mortars applied to the panels.

**Figure 8:**
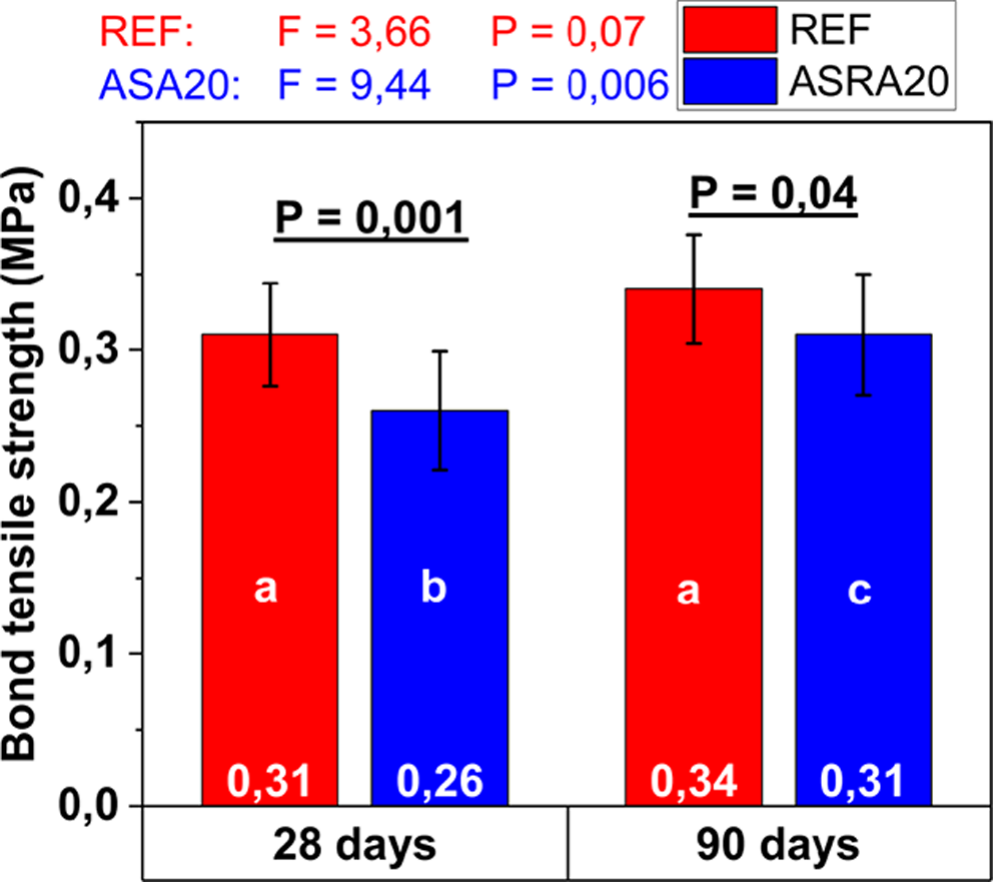
Bond tensile strength of REF and ASRA20 mortars at 28 and 90 days. Mean values ± standard deviation. Different lowercase letters indicate significant differences under the same variable. Source: Autor.

After analysis, it is possible to affirm that there is a statistical difference between the mortars and between the ages for the ASRA20 mortar, showing better performance compared to the REF mortar in the direct tensile bond strength test. At 28 days, the ASRA20 mortar did not reach the minimum tensile strength required for façade rendering, which must meet a minimum value of 0.30 MPa, as established by Brazilian Standard.^68^

It is important to highlight that the bond strength of coatings depends on a variety of factors that contribute to its development. In the specific case of this study, the following factors stand out: (a) the increased water-to-cement ratio, necessary to ensure adequate workability of the ASRA20 mortar, which tends to retain more moisture, initially weakening the matrix; (b) the slower progression of the hydration process, which may also be related to the intrinsic physical/chemical characteristics of the ash, as previously observed and discussed in the permeability evaluation.

On the other hand, at 90 days, a reduction in permeability and an increase in tensile strength were observed. As previously explained, the continued hydration of the cement allowed for the progressive filling of pores with hydration products, resulting in a gain in mechanical strength over time. Prolonged cement hydration significantly contributes to strength development at later ages, even in systems with a high water-to-cement ratio, such as those commonly used in mortar mixes in the metropolitan region of Belém, mainly due to the fine particle size of the local sand.^40,47^ Thus, to better understand the behavior of these materials, SEM and MIP analyses were employed to observe the microstructure and its relationship with mortar porosity.

In Figure 9A,C, two distinct morphologies are observed. The reference mortar presents a more porous and less dense structure. To complement this analysis, Mercury Intrusion Porosimetry (MIP) was performed. Figure 9B highlights a difference in the pore size distribution between the mortars, particularly within the characteristic range of 0.1 to 10 μm, with the reference mortar exhibiting smaller pore sizes. It is important to note that this pore structure is representative of the 90-day curing age, when cement hydration reactions are already at an advanced stage. This range falls within the so-called capillary pores, defined between 0.1 μm ≤ diameter ≤ 20 μm,^69^ which are crucial for mass transport in unsaturated porous media—typical of the building envelope systems where rendering mortars are applied.

**Figure 9:**
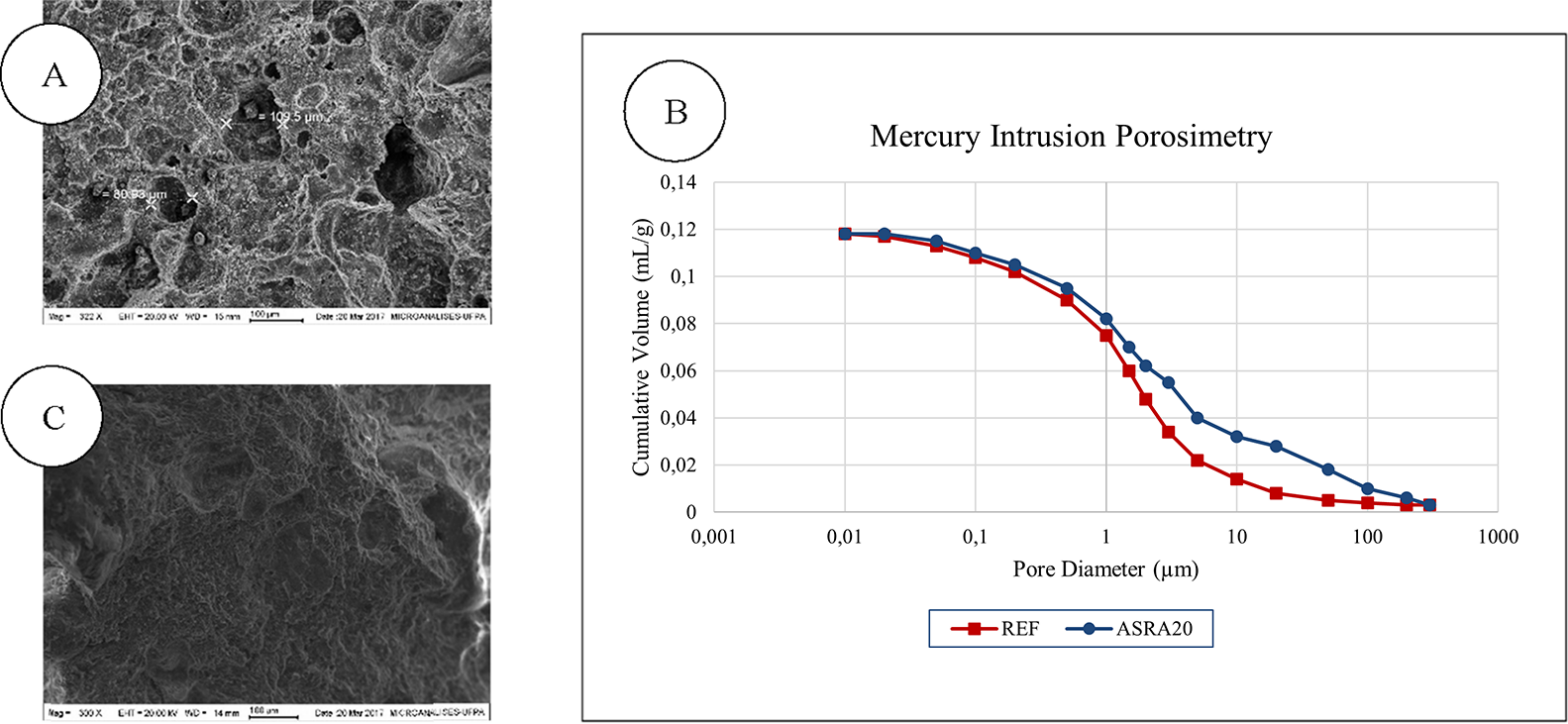
Microstructure obtained by scanning electron microscopy of REF and ASRA20 mortars, respectively (A and C); Direct tensile bond strength of REF and ASRA20 mortars at 28 and 90 days (B). Source: Autor.

The differences in pore structures reinforce that the reference mortar exhibits a denser “granular skeleton” compared to the ASRA20 mortar. This may be attributed to the residual ash mortar requiring a greater water demand due to its high specific surface area and more hydrophobic nature. As a result, cement hydration reactions, which develop over time, require a longer period to achieve strength levels that comply with standard specifications at 28 days.

The results showed that at this age, permeability and bond strength were below the normative limits. However, at 90 days, both properties reached satisfactory levels. The interaction between the mortars and the porous substrate led to mechanical “packing” due to water suction from the mortar interstices, enhancing the contact area and the bond between the solid components of the mix. Nevertheless, in the case of the açaí stone ash mortar, a longer curing period was required to meet normative standards.^38,70,71^

In parallel with this work, the incorporation of agro-industrial residues into rendering mortars has proven to be promising. Examples include the use of charcoal residues^72^ and chemically treated coffee,^73^ both of which yielded relevant results. Furthermore, studies with vermiculite-based mixtures^74^ and eco-mortars incorporating incineration ash^75^ have demonstrated improvements in both mechanical performance and durability. These investigations highlight the potential of waste valorization in the construction sector, while also indicating the need for preprocessing steps (such as drying, grinding, and particle size classification) to ensure reproducibility.

In the specific case of açaí seed residual ash (ASRA), large-scale use still depends on practical aspects related to collection and logistics in the Amazon region, where seasonality and the dispersion of agro-industries pose additional challenges. Within this context, the present study emphasizes its relevance and originality by going beyond laboratory testing to assess ASRA performance under real application conditions, thereby contributing to the advancement of agro-industrial residue integration into the construction value chain.

## 4 Conclusions

Based on the experimental results and analyses conducted, the following conclusions can be drawn:

- The production of an ash with an amorphous structure would be ideal to maximize pozzolanic activity; however, the uncontrolled combustion process limited the development of such phases. Consequently, the açaí seed residual ash (ASRA) acted primarily as a filler material, contributing through its physical packing effect rather than chemical reactivity.
- In the fresh state, ASRA incorporation increased the water demand required to achieve workability, which in turn led to higher drying shrinkage. This effect was directly proportional to the replacement level.
- Among the tested formulations, the ASRA20 mortar provided the most balanced performance, combining adequate consistency, mechanical strength, and reduced water absorption, even outperforming the reference mortar in selected properties.
- In painel applications, ASRA20 exhibited satisfactory scratch resistance, surface cohesion, and progressive hardness gain at later ages (90 days). Nonetheless, the direct tensile bond strength at 28 days was slightly below the normative minimum (0.30 MPa. This limitation was mitigated at 90 days, when the bond strength surpassed the required threshold owing to continued hydration.

From a broader perspective, the study demonstrates that ASRA, when properly processed, represents a promising alternative material for rendering mortars. Its use contributes to

1. Environmental benefits — reducing natural sand consumption and promoting the valorization of an abundant agro-industrial residue from the Amazon region.
2. Technical potential — improving particle packing, enhancing certain mechanical properties, and ensuring adequate performance in real-scale applications.
3. Circular economy alignment — transforming a low-value residue into a functional raw material for construction, thereby fostering regional sustainability strategies.

Future work should focus on:

- Developing controlled combustion, thermal, or chemical activation methods to increase reactivity;
- Optimizing grinding and classification processes to refine filler effects and reduce porosity;
- Investigating admixture combinations to mitigate water demand and enhance early age adhesion;
- Assessing long-term durability under aggressive exposures (moisture cycles, carbonation, sulfate attack, thermal variations);
- Conducting techno-economic and life cycle assessments (LCA) to support scalability and industrial adoption.

In conclusion, despite the need for further standardization and optimization, the findings underscore the potential of açaí seed residual ash as a sustainable raw material in rendering mortars. Its adoption may significantly reduce environmental impacts associated with natural aggregate extraction and contribute to the development of greener construction practices, particularly relevant within the Amazonian context and other regions with similar agro-industrial waste challenges.
